# High pre-exposure prophylaxis uptake and early adherence among men who have sex with men and transgender women at risk for HIV Infection: the PrEP Brasil demonstration project

**DOI:** 10.7448/IAS.20.1.21472

**Published:** 2017-04-06

**Authors:** Brenda Hoagland, Ronaldo I. Moreira, Raquel B. De Boni, Esper G. Kallas, José Valdez Madruga, Ricardo Vasconcelos, Silvia Goulart, Thiago S. Torres, Luana M. S. Marins, Peter L. Anderson, Paula M Luz, Iuri da Costa Leite, Albert Y. Liu, Valdilea G. Veloso, Beatriz Grinsztejn, for the PrEP Brasil Study Team

**Affiliations:** ^a^ FIOCRUZ, Instituto Nacional de Infectologia Evandro Chagas, Rio de Janeiro, Brazil; ^b^ School of Medicine, Universidade de São Paulo, Brazil; ^c^ School of Medicine, Universidade de São Paulo, São Paulo, Brazil; ^d^ Skaggs School of Pharmacy and Pharmaceutical Sciences, University of Colorado Denver, Aurora, CO, USA; ^e^ San Francisco Department of Public Health, Bridge HIV, San Francisco, CA, USA

**Keywords:** Pre-exposure prophylaxis, HIV prevention, MSM, transgender women, PrEP uptake, PrEP adherence, DRUG Levels

## Abstract

**Introduction**: The efficacy of pre-exposure prophylaxis (PrEP) in preventing sexual acquisition of human immunodeficiency virus (HIV) is well established. Little is known about the feasibility of PrEP implementation in middle-income settings with concentrated epidemics among men who have sex with men (MSM) and transgender women (TGW).

**Methods**: PrEP Brasil is a prospective, multicentre, open-label demonstration project assessing PrEP delivery in the context of the Brazilian Public Health System. HIV-uninfected MSM and TGW in 3 referral centres in Rio de Janeiro and São Paulo were evaluated for eligibility and offered 48 weeks of daily emtricitabine/tenofovir for PrEP. Concentrations of tenofovir diphosphate in dried blood spot samples (DBS) at week 4 after enrolment (early adherence) were measured. Predictors of drug levels were assessed using ordinal logistic regression models considering the DBS drug level as a 3 level variable (<350 fmol/punch, ≥350–699 fmol/punch and ≥700 fmol/punch).

**Results**: 1,270 individuals were assessed for participation; *n* = 738 were potentially eligible and *n* = 450 were offered PrEP (PrEP uptake was 60.9%). Eligible but not enrolled individuals were younger, had lower HIV risk perception and had lower PrEP awareness. At week 4, 424 participants (of the 450 enrolled) had DBS TFV-DP concentrations, 94.1% in the protective range (≥350 fmol/punch, consistent with ≥2 pills per week), and 78% were in the highly protective range (≥700 fmol/punch, ≥4 pills per week). Participants with ≥12 years of schooling had 1.9 times the odds (95%CI 1.10–3.29) of a higher versus lower drug level than participants with <12 years of schooling. Condomless receptive anal intercourse in the prior 3 months was also associated with higher drug levels (adjusted OR = 1.78; 95% CI 1.08–2.94).

**Conclusions**: The high uptake and early adherence indicate that PrEP for high-risk MSM and TGW can be successfully delivered in the context of the Brazilian Public Health System. Interventions to address disparities on PrEP awareness and HIV risk perception among the younger and less educated are urgently needed in order to maximize the impact of this prevention strategy on the reduction of HIV infection among MSM and TGW in Brazil.

## Introduction

The effectiveness of oral pre-exposure prophylaxis (PrEP) using tenofovir/emtricitabine (TDF/FTC) for prevention of sexually acquired human immunodeficiency virus (HIV) infection for men who have sex with men (MSM) and transgender women (TGW) has been demonstrated in randomized trials and open-label studies [1–5]. PrEP effectiveness results from achieving protective drug levels in the blood which is directly related to the level of adherence to the medication use [1,6,7]. In the iPrEx study, protection was estimated to be over 90% in those with detectable drug levels in their blood [1], with pharmacokinetic modelling suggesting that efficacy reaches 96 and 99% with dosing of four and seven days per week, respectively [8]. Subsequent results from the open-label extension of the iPrEx study estimated that 90% protection was achieved with 2–3 drug doses/week and that 4 or more doses/week were highly protective against HIV infection [9]. Similarly, Cottrell et al., in a study that combined an *in vitro* efficacy target with mucosal tissue pharmacokinetic data and mathematical modelling, also found that 2 doses/week resulted in effective colorectal concentrations in >95% of the population [10]. Drug levels consistent with the intake of 4 or more pills/week were observed in only about one-third of study follow-up visits in iPrEx OLE [11], corroborating that adherence is a significant challenge to PrEP effectiveness.

International guidelines were released recommending PrEP for MSM and TGW at “substantial risk” for acquiring HIV [12,13]. However, PrEP is not widely available and the feasibility of this prevention strategy in real world settings from low- and middle-income countries is unknown. As of December 2016, no country in Latin America had implemented PrEP as a public health policy. In Brazil, specifically, PrEP can only be obtained in the context of research or through a commercial vendor. The HIV epidemic in Brazil persists unabated in the MSM and TGW populations [14]. While HIV prevalence among the general population is 0.6%, in MSM it reaches 14.2% [14]. TGW represent a smaller population than MSM, nevertheless they have extremely elevated HIV infection rates [15].

PrEP Brasil is a multicentre, open-label PrEP demonstration project to assess the uptake, adherence, safety, and feasibility of PrEP implementation provided at no cost to high-risk MSM and TGW in the context of the Brazilian public health system. In this manuscript, we describe PrEP uptake and early adherence [16] assessed by TDF/FTC drug levels (i.e. tenofovir-diphosphate and FTC-triphosphate) measured in dried blood spots (DBS) at week 4 after enrolment and its associated factors in the PrEP Brasil demonstration study. Additionally, we describe baseline demographic and risk characteristics of the study population.

## Methods

### Study design, sites, and study population

PrEP Brasil is a 48-week prospective, longitudinal, open-label demonstration study assessing PrEP delivery at three reference centres for HIV prevention and care in Rio de Janeiro (RJ) and São Paulo (SP), the two cities in Brazil with the highest burden of HIV cases [17]. Study sites are Fundação Oswaldo Cruz (FIOCRUZ) in RJ, Universidade de São Paulo (USP) and Centro de Referência e Treinamento em DST e AIDS (CRT-SP), both in SP. All the three sites have outstanding expertise in providing HIV prevention and care services in the context of Brazilian Public Health System (SUS). In addition, these sites conduct research in the HIV field. In RJ, individuals seeking testing at Arco Iris, a lesbian, gay, bisexual, transgender (LGBT) non-governmental organization (NGO), and at a mobile testing unit located at a LGBT-friendly venue were also assessed for potential eligibility for PrEP Brasil and subsequently referred to FIOCRUZ for screening. Social media and other media were used by the 3 sites to advertise the project and a website was constructed (www.prepbrasil.com.br). Individuals were assessed for participation from 1 April 2014 to 28 July 2015, enrolled from 4 June 2014 to 28 July 2015, and collection of week 4 results ended in 24 August 2015.

### Eligibility criteria

Eligibility criteria included ≥18 years of age, male sex at birth, residence in RJ or SP, reporting sex with male or TGW and any of the following sexual risk criteria in the prior 12 months: ≥2 episodes of condomless anal sex, ≥2 episodes of anal sex with an HIV-infected partner, or history of STD diagnosis. Individuals were ineligible, if presenting with any of the following criteria: a positive HIV rapid test, creatinine clearance <60 ml/min, proteinuria (urine dipstick 1+ or more), a positive Hepatitis B surface antigen (HBsAg) serology test, a severe medical comorbidity, use of antiretrovirals (ARV), interferon or interleukin, or failure to provide contact information or return for enrolment within 45 days of the screening visit.

### Study procedures

Individuals either self-referred or clinic referred to take part in PrEP Brasil when searching for HIV testing, post exposure prophylaxis (PEP) or health services.

At the pre-screening visit, a self-answered structured interview using tablets assessed demographics, HIV risk perception, sexual risk entry criteria, self-reported HIV serostatus, awareness and willingness to use PrEP and other HIV prevention methods. HIV rapid testing was offered [18]. In addition, individuals with a negative HIV rapid test who reported condomless anal intercourse in the last 30 days were offered pooled or individual HIV RNA testing (pooled RNA in RJ and individual RNA in SP) to diagnose acute HIV infection. Potentially eligible individuals were invited to participate in PrEP Brasil. There was no pre-determined timeframe between pre-screening and screening visits, but individuals were encouraged to show-up for screening within one week of pre-screening. Refusal reasons were noted.

At the screening visit, participants were informed about study procedures and visit schedule. Laboratory assessments included HIV rapid testing, pooled or individual HIV RNA, HBsAg, hepatitis C antibody, syphilis, creatinine clearance and proteinuria (urine dipstick 1+ or more). Potential participants received a thorough explanation of the potential risks and benefits of FTC/TDF for PrEP as well as the importance of study drug adherence and risk reduction counselling.

The enrolment visit of eligible individuals was scheduled within 45 days of the screening visit. At this visit, HIV rapid test and pooled or individual HIV RNA were performed, as well as creatinine clearance and proteinuria (urine dipstick 1+ or more). A rectal sample was collected for *Chlamydia trachomatis* (CT) and *Neisseria gonorrhoea* (NG) detection. Participants answered a computer-assisted self-interview (CASI) including demographics, sexual and drug-use behaviour questions. Eligible individuals were offered daily oral PrEP with TDF/FTC.

At week 4 visit, HIV testing (rapid test and pooled or individual HIV RNA), creatinine clearance and proteinuria (urine dipstick 1+ or more), clinical evaluation and DBS collection for tenofovir-diphosphate (**TFV-DP)** and FTC-triphosphate (**FTC-DP)** assessments were performed. Individuals could formally refuse to participate in any of the visits, or simply not show up in the subsequent scheduled visit.

### Laboratory procedures

HIV testing was performed following the Brazilian Ministry of Health algorithm [19]. Briefly, two different HIV rapid tests were performed when the first test was positive. If the second test was positive, individuals were considered HIV infected; if the second test was negative, individuals were considered indeterminate. Individuals with a first negative test were classified as HIV uninfected. Pooled or individual HIV RNA was performed at screening, enrolment and at each study visits thereafter.

A rapid plasma reagin (RPR) test was performed for syphilis screening; positive results were confirmed using a microhemagglutination assay for *Treponema pallidum* (MHA-TP). Active/recent syphilis was defined as titres ≥1/8 and a positive MHA-TP (WAMA Diagnóstica, SP). Rectal *Chlamydia trachomatis* (CT) and *Neisseria gonorrhoea* (NG) detection was performed using the Abbott Real Time platform and the NG/CT Amplification Reagent Kit (Abbott Molecular, Des Plains, IL). All indeterminate results for rectal CT/NG were repeated using the same tests on the same sample. If the repeated test was conclusive, the results were reported accordingly. If remained indeterminate, results were reported as negative. All rectal CT and NG samples were processed at the FIOCRUZ Laboratory.

## Measures

### Socio-demographic

Age was categorized in 3 strata: 18–24 years; 25–34 years and ≥35 years; skin colour/race (white, black, mixed-black, native, Asian) were categorized in white, black and mixed; schooling was dichotomized in <12 years and ≥12 years (12 years is equivalent to completing high school education in Brazil) and the sites were grouped accordingly to geographical locations (RJ and SP). Gender was considered as “Male” and TGW. Housing situation, assessed at enrolment using CASI, was dichotomized as rent/own housing or other (living with friends or family, living in public housing). Individuals were asked if they had a steady partner and answered yes/no at their own discretion.

### Sexual behaviour and sexually transmitted diseases

All variables related to sexual behaviour refer to the prior 3 months and were assessed at enrolment using CASI; these questions refer to the participant’s prior 3 partners. Those who responded that any of the 3 prior partners was a “client” were considered as having sex with clients. Similarly, dichotomous variables were created for condomless receptive anal intercourse and for sex with an HIV-positive partner. The number of male, TGW and female partners was assessed, as well as the possible sexual roles with these partners (“Insertive”, “Receptive” or “Both”). A dichotomous variable “STD diagnosis” was created considering any positive laboratorial diagnosis for syphilis, gonorrhoea or chlamydia.

### Substance use, mental health, and hormone use

Binge drinking [20] was evaluated with the question “In the last 3 months, did you drink 5 or more drinks in a couple of hours?” “Any illicit drug” considered the use of any of the following: marijuana, stimulants (cocaine, crack, amphetamines), hallucinogens (solvents, LSD, ketamine) and opioids (heroin, methadone), which were shown in a pre-defined list of all substances participants could have used in the prior 3 months. The list also included non-medical use of tranquilizers and erectile dysfunction drugs. Use of any injectable substance (IDU) was also assessed. Depression was screened using the Patient Health Questionnaire-2 (PHQ-2) using a score ≥3 as the cut-off for a positive screen [21,22]. For TGW, hormone use was captured as concomitant medication use at the enrolment visit.

### Risk perception

In the pre-screening interview, risk perception was assessed by the question “What is your chance of getting HIV in the next year?” with possible options dichotomized into Low (None/Low) and High (Some/High/Certainly). Additionally individuals were asked about previous HIV testing in the prior year (Yes/No). At enrolment, the number of HIV tests (with possible answers 0, 1–3, >3) in the last 12 months was considered as a proxy of risk perception, as well as the reported use of post-exposure prophylaxis (PEP).

### Safety monitoring

All adverse events were graded using the Division of AIDS (DAIDS) Adverse Event Grading Table [23]. Clinical symptoms, including gastrointestinal (GI) symptoms were evaluated at week 4 and were dichotomized (Yes/No) indicating the presence of at least one of the following: abdominal pain, diarrhoea, flatulence, nausea and vomiting. Proteinuria and creatinine clearance were assessed at screening, enrolment and week 4 visits.

### Main outcome

TFV-DP and FTC-TP were assessed for all study participants at week 4 in DBS cards using liquid chromatography and mass-spectroscopy (LC/MS/MS). Our main outcome of interest was early adherence as measured by TFV-DP drug levels at week 4. In addition, descriptive results of the FTC-TP levels are presented. DBS samples were stored at −20ᵒC within 24 h of collection and shipped on dry ice to the University of Colorado Antiviral Pharmacology Laboratory after study enrolment was finalized. Three millimetres punches were extracted and analyzed for TFV-DP and FTC-TP by LC/MS/MS, as previously described [2,24,25]. Week 4 values were used to estimate steady-state values based on a 17-day half-life for interpretation. For the purposes of the statistical analysis, a 3-level ordinal variable classifying participants as TFV-DP <350fmol/punch (<2 doses/week), ≥350–699 fmol/punch (2–3 doses/week-protective range) or ≥700 fmol/punch (≥4 doses/week; highly protective range) was created. This categorization of TFV-DP concentrations was used in the iPrEx Open Label Extension [2] and derived from previous pharmacokinetic modelling studies [8].

### Statistical analysis

Variables describing the characteristics of potentially eligible individuals (enrolled and not enrolled), as well as enrolled participants by site location are presented in terms of absolute numbers and proportions, when categorical, and median and interquartile range when continuous. Distributions were compared using chi-square test, Fisher exact test or Kruskal–Wallis statistics, as appropriate. PrEP uptake was defined as the number of participants enrolled divided by the number of potentially eligible participants at the pre-screening visit minus the clinical ineligible participants (at screening and enrolment visits) [26]. Factors associated with PrEP uptake were evaluated using logistic regression model while predictors of drug levels were assessed using ordinal logistic regression models considering the DBS drug level as a 3 level variable (<350 fmol/punch, ≥350–699 fmol/punch and ≥700 fmol/punch). Only variables statistically significant at 5% in the unadjusted models were kept in the adjusted models. The use of the ordinal logistic regression model requires that the effect of each predictor is the same for different logit function, that is, the odds ratio comparing the odds of high drug levels (≥4 doses) to the odds of medium-low drug levels (2–3 doses and <2 doses) is the same as that obtained when the comparing the odds of high-medium drugs levels (≥4 doses and 2–3) to the odds of low drug levels (<2 doses). This proportional odds assumption was evaluated using the SCORE test [27]. The results of the logistic ordinal model were then reported in terms of odds ratio (OR), which can be interpreted as the effect of the variable on the odds of being in a higher versus lower category of drug levels. The association of GI symptoms and drug levels at week 4 was evaluated using ordinal logistic regression models [28]. Additionally, to explore possible effects of hormone use on TFV-DP levels among TGW, two modelling approaches were used: (1) a logistic regression model with ≥4 doses/week as the outcome and use/non-use of hormone as the dichotomous explanatory variable, (2) a linear regression model with TFV-DP concentration as the continuous outcome variable and use/non-use of hormone as the dichotomous explanatory variable. Analyses were performed using PROC GENMOD available in the Software SAS [27].

### Ethical aspects

Institutional review boards at each site approved the study and all study participants signed an informed consent form at pre-screening and screening visits.

## Results

**Figure 1.**
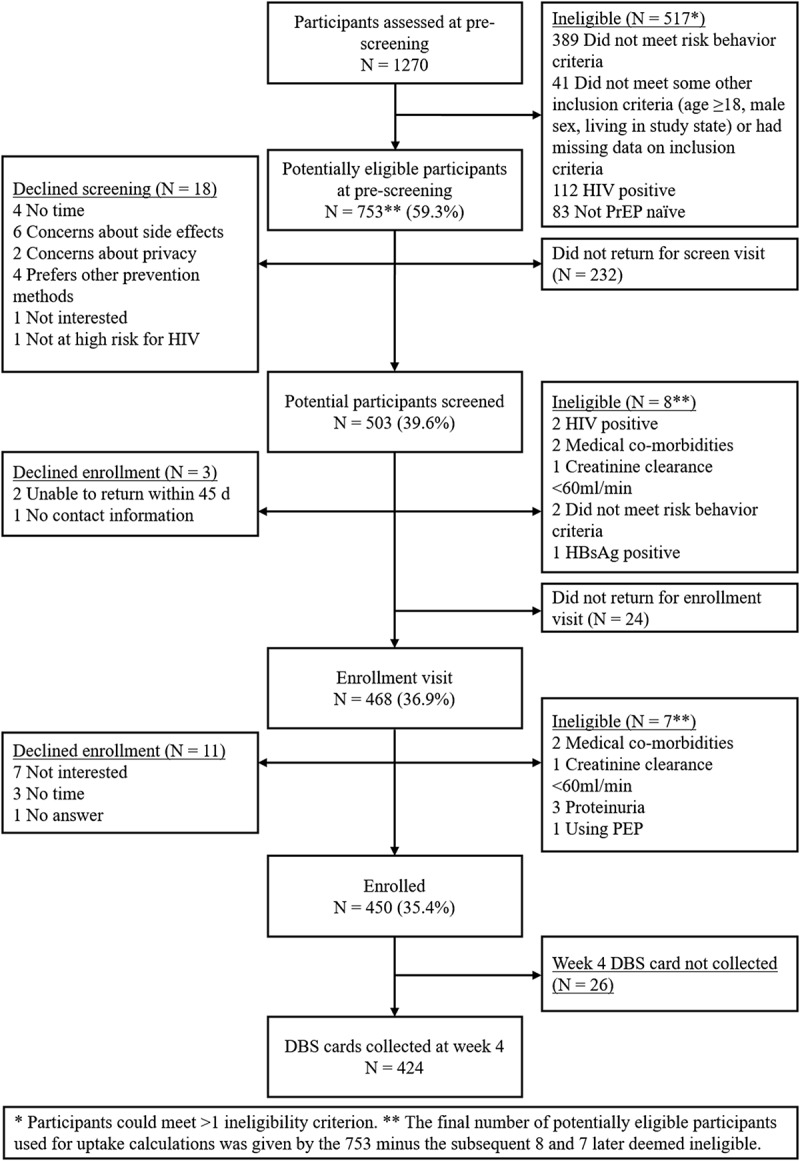
Inclusion flowchart - PrEP Brasil, 2014-15.

Overall, 1270 individuals were assessed at the pre-screening visit at the 3 sites. Of these, 517 (40.7%) were ineligible, and 753 (59.3%) were potentially eligible and invited to participate in PrEP Brasil. Among the 18 that refused to participate, the main refusal reasons were lack of time and fear of side effects. In total, 232 (232/753; 30.8%) potentially eligible individuals who accepted to participate did not show up for the screening visit and 18 declined, leading to 503 (503/1270; 39.6%) screened individuals. There were 8 screening failures, two of which were acute HIV infections. Thus, the prevalence of acute infection at the screening visit was 0.4% (2/503 × 100). In addition, one individual at pre-screening had a diagnosis of acute HIV infection (negative HIV rapid test and a detectable HIV RNA). Twenty-four individuals did not show up, 3 declined to be enrolled and 468 were evaluated for enrolment. Of these, 11 declined enrolment during the visit and 7 individuals were deemed ineligible. In the end, 450 participants were enrolled (450/1270; 35.4%) (Figure 1). The final number of potentially eligible participants (738) used for PrEP uptake calculations was given by the initial 753 minus the subsequent 15 participants who were subsequent deemed ineligible during screening and enrolment visits.

PrEP uptake was 60.9% (450 enrolled/738 potentially eligible). Most of the potentially eligible individuals were self-referred (*n* = 310/559 available answers; 55.5%) and 249 were clinic referrals (249/559; 44.5%). Table 1 depicts the characteristics of potentially eligible individuals stratified by enrolment status (uptake). Variables significantly associated with showing up to the screening visit were the same as those associated with uptake (Supplementary Table 1). Compared to those enrolled, a higher proportion of eligible but not enrolled individuals was younger, less educated, more frequently self-defined as of mixed race, had lower HIV risk perception, lower prior HIV testing rates, lower PrEP awareness and was less likely to report anal sex with partners of unknown HIV status (all *p* < 0.05). According to the adjusted logistic regression model, factors independently associated with uptake were site location (aOR = 5.03; 95% CI: 3.37–7.50, SP compared to RJ), perceiving a 50–100% chance of getting HIV in the next year (aOR = 1.44; 95% CI: 1.03–2.02) and having prior PrEP awareness (aOR = 2.19; 95% CI: 1.52–3.16).

**Table 1.** Characteristics of potentially eligible individuals by enrolment status, PrEP Brasil

	Overall	Uptake		Unadjusted		Adjusted	
	Potentially eligible individuals N=738* (%)	Enrolled N=450 (%)	Not enrolled N=288(%)	OR	p-value	aOR	p-value
**Site Location**							
RJ	413 (56.0)	180 (40.0)	233 (80.9)	1	-	1	-
SP	325 (44.0)	270 (60.0)	55(19.1)	6.35(4.48-9.01)	<0.01	5.03 (3.37-7.50)	<0.01
**Age**							
18-24 years	210 (28.5)	113 (25.1)	97 (33.7)	0.54 (0.36-0.82)	<0.01	0.78 (0.49-1.25)	0.30
25-34 years	348 (47.2)	214 (47.6)	134 (46.5)	0.74(0.51-1.08)	0.12	0.77 (0.50-1.19)	0.24
>=35 years	180 (24.3)	123 (27.3)	57 (19.8)	1	-	1	-
**Schooling**							
< 12 years	227 (30.8)	115 (25.6)	112 (38.9)	1	-	1	-
≥ 12 years	511 (69.2)	335(74.4)	176 (61.1)	1.85(1.35-2.55)	<0.01	0.89 (0.60-1.31)	0.56
**Color/Race**							
White	358 (48.5)	243 (54.0)	115 (40.0)	1	-	1	-
Black	102 (13.8)	57 (12.7)	45 (15.6)	0.60 (0.38-0.94)	0.02	1.26 (0.75-2.12)	0.37
Mixed**	272 (36.9)	145 (32.2)	127 (44.1)	0.54(0.39-0.75)	<0.01	0.98 (0.66-1.43)	0.90
**Gender**							
Male	703 (95.3)	425 (94.4)	278 (96.5)	1	-	-	-
TGW	35 (4.7)	25 (5.6)	10 (3.5)	1.64 (0.77-3.46)	0.20	-	-
**Steady Partner**							
Yes	376 (51.9)	233 (53.3)	143 (49.8)	1.15 (0.85-1.55)	0.36	-	-
No	348 (48.1)	204 (46.7)	144 (50.2)	1	-	-	-
**Perceived likelihood of getting HIV in the next year**							
0-25%	377 (52.1)	205 (46.9)	172 (59.9)	1	-	1	-
50-100%	347 (47.9)	232 (53.1)	115 (40.1)	1.69 (1.25-2.29)	<0.01	1.44 (1.03-2.02)	0.03
**Previous HIV test (12 months)**							
Yes	538 (74.3)	359 (82.2)	179 (62.4)	2.78 (1.97-3.91)	<0.01	1.36 (0.91-2.03)	0.13
No	186 (25.7)	78 (17.8)	108 (37.6)	1	-	1	-
**Prior PrEP awareness**							
Yes	466 (64.1)	328 (74.5)	138 (48.1)	3.16 (2.31-4.34)	<0.01	2.19 (1.52-3.16)	<0.01
No	261 (35.9)	112 (25.5)	149 (51.9)	1	-	1	-
**# male condomless a-l sex partners (last 12 months)**							
<2	252 (34.8)	143(32.7)	109 (38.0)	1	-	-	-
2 or more	472 (65.2)	294(67.3)	178 (62.0)	1.26 (0.92-1.72)	0.15	-	-
**A-l sex with HIV-positive partners**							
Yes	360 (49.7)	223(41.0)	137 (47.8)	1.38 (0.90-2.13)	0.14	-	-
No	97 (13.4)	47 (10.8)	50 (17.4)	1	-	-	-
I do not know	267 (36.9)	167(38.2)	100 (34.8)	1.42 (0.91-2.22)	0.12		
**STD history (12months)**							
Yes	139 (19.2)	87 (19.9)	52 (18.1)	1.12 (0.77-1.64)	0.55	-	-
No	585 (80.8)	350(80.1)	235 (81.9)	1	-	-	-

TGW= Transgender women, STD =Sexually transmitted diseases, RJ = Rio de Janeiro, SP= São Paulo.

*For 16 participants, data obtained by ACASI interview in pre screening were not properly synchronized resulting in missing values​​. Missing information were fully recovered for Site, Age, Schooling and Race, and partially recovered for Gender and Prior PrEP awareness.** Category composed of “yellow and indigenous”

From the 450 enrolled participants, 265 (76.8%; 265/345 available answers) were self-referred and 80 (23.2%; 80/345) were clinic referrals. Baseline characteristics for the 450 enrolled participants according to site location are shown in Table 2. Median age was 30 years (interquartile range (IQR) 24–35 years), the majority self-referred as white (*n* = 243, 54%) and reported their living situation as rent or own housing (*n* = 289, 64.2%). Forty per cent (*n* = 180) of the enrolled individuals were from RJ, most had ≥12 years of education (*n* = 335, 74.4%), and reported a steady partner (*n* = 254, 56.4%). Overall, 94.7% of the participants were male (89.9% self-identified as gay) and 5.3% were TGW. All demographic characteristics, except reporting a steady partner, were significantly different (*p* < 0.05) between site locations.

**Table 2.** Participants characteristics at enrolment by site location. PrEP Brasil-2015

Participant’s characteristics	Total	RJ	SP	Chi-Square
**Overall**	**450^1^**	**180 (40.0%)**	**270 (60%)**	<0.01
**Socio-demographics^2^**				
**Age**				0.02
18–24 years	113	58 (32.2%)	55 (20.4%)	
25–35 years	214	76 (42.2%)	138 (51.1%)	
≥35 years	123	46 (25.6%)	77 (28.5%)	
**Colour/Race**				<0.01
White	243	60 (33.5%)	183 (68.8%)	
Black	57	41 (22.9%)	16 (6.0%)	
Mixed Race	145	78 (43.6%)	67 (25.2%)	
**Schooling**				<0.01
<12 years	115	81 (45.0%)	34 (12.6%)	
≥12 years	335	99 (55.0%)	236 (87.4%)	
**Gender**				<0.01
Male	425	161 (89.4%)	265 (97.8%)	
Trans women	25	19 (10.6%)	6 (2.2%)	
**Housing situation**				<0.01
Rent or own housing	289	98 (55.7%)	191 (71.5%)	
Other (live with friends/family, live in public housing)	154	78 (44.3%)	76 (28.5%)	
**Steady partner**				0.51
Yes	254	105 (58.3%)	149 (55.2%)	
No	196	75 (41.7%)	121 (44.8%)	
**Sexual behaviour in last 3 months**				
**Had sex with client**				0.63
Yes	27	12 (6.7%)	15 (5.6%)	
No	423	168 (93.3%)	255 (94.4%)	
**Unprotected receptive anal intercourse**				0.28
Yes	201	86 (47.8%)	115 (42.6%)	
No	249	94 (52.2%)	155 (57.4%)	
**Sex with HIV-positive partners**				0.11
Yes	223	80 (46.5%)	143 (54.4%)	
No	212	92 (53.5%)	120 (45.6%)	
**Median Number of male partners (IQR)**	3 (1–10)	3 (1–10)	4 (1–10)	0.22 ^3^
**Sexual role with male partners**				0.29
Insertive	112	41 (23.0%)	71 (26.6%)	
Receptive	49	16 (9.0%)	33 (12.4%)	
Both	284	121 (68.0%)	163 (61.0%)	
**Substance use and mental health**				
**Binge drinking**				0.91
Yes	266	107 (59.4%)	159 (58.9%)	
No	184	73 (40.6%)	111 (41.1%)	
**Any illicit drug use in past 3 months**				0.05
Yes	134	44 (24.7%)	90 (33.5%)	
No	313	134 (75.3%)	179 (66.5%)	
**Marijuana**				0.19
Yes	128	45 (25.0%)	83 (30.7%)	
No	322	135 (75.0%)	187 (69.3%)	
**Stimulants (cocaine, crack, amphetamines)**				0.01
Yes	62	16 (8.9%)	46 (17.0%)	
No	388	164 (91.1%)	224 (83.0%)	
**Hallucinogens (solvents, LSD, ketamine)**				0.45
Yes	38	13 (7.2%)	25 (9.3%)	
No	412	167 (92.8%)	245 (90.7%)	
**Tranquilizers**				0.44
Yes	30	10 (5.6%)	20 (7.4%)	
No	420	170 (94.4%)	250 (92.6%)	
**Erectile dysfunction drugs**				0.10
Yes	51	15 (8.3%)	36 (13.3%)	
No	399	165 (91.7%)	234 (86.7%)	
**Depression PHQ score**				0.62
PHQ-2 score ≥ 3	27	12 (6.7%)	15 (5.6%)	
PHQ-2 score < 3	422	167 (93.3%)	255 (94.4%)	
**STD diagnosis**				0.87
Yes	89	35 (19.7%)	54 (20.3%)	
No	355	143 (80.3%)	212 (79.7%)	
**Active/recent syphilis^2^**				0.94
Yes	43	17 (9.6%)	26 (9.8%)	
No	401	161 (90.4%)	240 (90.2%)	
**Chlamydia**				0.05
Yes	36	9 (5.1%)	27 (10.2%)	
No	408	169 (94.9%)	239 (89.8%)	
**Gonorrhoea**				0.33
Yes	22	11 (6.2%)	11 (4.1%)	
No	422	167 (93.8%)	255 (95.9%)	
**Risk perception**				
**Number of HIV tests in 12 months prior inclusion**				<0.01
0	76	52 (32.7%)	24 (10.4%)	
1–3	235	91 (57.2%)	144 (62.3%)	
>3	79	16 (10.1%)	63 (27.3%)	
**PEP in 12 months prior inclusion**				0.34
Yes	91	33 (20.9%)	58 (25.0%)	
No	299	125 (79.1%)	174 (75.0%)	
**Any GI symptoms^2^**				0.01
Yes	178	40 (24.1%)	138 (53.5%)	
No	246	126 (75.9%)	120 (46.5%)	

TGW= Transgender women, STD =Sexually transmitted diseases, RJ= Rio de Janeiro, SP= São Paulo, PHQ= Patient Health Questionnaire, GI= Gastrointestinal. PEP = Post exposure prophylaxis. ^1^There was missing information for: Housing situation (*n* = 7), Sexual role with male (*n* = 5), Any illicit drug use in last 3 months (*n* = 3), STDs (*n* = 6), Number of HIV tests in 12 months prior inclusion (*n* = 60), PEP in 12 months prior inclusion (*n* = 60). 5 individuals who reported yellow/indigenous are not included in this table. ^2^Socio-demogrphics, except “Housing situation”, were assessed at pre-screening visit. Syphilis was assessed at screening visit and GI symptoms, at week 4. All other variables are from enrolment visit. ^3^Kruskal-Wallis Test.

The median number of male anal sex partners in the previous 3 months was 3 (IQR 1–10) and none of the participants reported sex with TGW or women. Most (*n* = 284, 63.1%) reported both receptive and insertive anal sex and 44.7% (*n* = 201) reported condomless receptive anal intercourse in previous 3 months and these behaviours were not significantly different across sites. Any STD at screening/enrolment was diagnosed in 20% (89/444) of the participants: 9.6% (43/444) with active/recent syphilis, 4.9% with rectal gonorrhoea (22/444) and 8.1% (36/444) with rectal chlamydia. Any illicit drug use in the prior 3 months was reported by 29.9% (134/447), and the most frequent drugs reported were marijuana (128/450, 28.4%) and stimulants (62/450, 13.7%). IDU was reported by 1.1% (5/450). Stimulant use was more prevalent in SP (*p* = 0.01).

From the 450 enrolled participants, 26 (5.8%) did not collect DBS at week 4. For the 424 (94.2%) participants who had DBS samples at week 4: 5.9% (*n* = 25) had TFV-DP level consistent with <2 doses/week, 15.6% (*n* = 66) with 2–3 doses/week and 78.5% (*n* = 333) were in the highly protective range, corresponding to ≥4 doses/week. Almost 90% of the participants (379/424) had FTC-TP detectable concentrations at week 4, indicating dosing within the last 48 h [24]. Table 3 presents participant’s characteristics according to drug levels, the unadjusted and adjusted factors associated with higher drug levels. Results from the unadjusted models showed that the assumption of proportional odds was upheld for all variables as the *p*-values are higher than 0.05. Four variables were statistically significant at 5% (*p* < 0.05): site location, colour/race, schooling and condomless receptive anal intercourse in the prior 3 months. The odds of achieving a higher versus lower drug level by site location were attenuated in the adjusted model (aOR = 1.66 for SP compared with RJ; 95% CI: 0.97–2.84). The loss of significance of site location was explained by the inclusion of schooling in the model, as the proportion of participants with ≥12 years schooling in SP (87.4%) was much higher than in RJ (55%). In fact, in the adjusted model schooling was the strongest predictor of the drug level: participants with ≥12 years had 1.9 times the odds of a higher versus lower drug level than participants with <12 years of schooling. Finally, condomless receptive anal intercourse in the prior 3 months was also associated with higher drug levels (aOR = 1.78; 95% CI: 1.08–2.94).

**Table 3.** Unadjusted and adjusted odds ratios and 95% confidence interval obtained from the ordinal logistic model for the level of TFV-DP according to a set of participant’s characteristics aOR = adjusted odds ratio. PrEP Brasil, 2015.

		Level of TFV-DP (%)			Unadjusted		Adjusted	
Characteristic	Total	<2 doses/week *N*(%)	2–3 doses/week *N*(%)	≥4 doses/week *N*(%)	OR (95%CI)	*p*-Value	aOR (95%CI)	*p*-Value
**Overall**	**424**	25 (5.9)	66 (15.6)	333 (78.5)	NA	NA	NA	NA
**Socio-demographics^1^**								
**Site location**								
RJ	169	16 (9.5)	33 (19.5)	120 (71.0)	1.00	NA	1.00	NA
SP	255	9 (3.5)	33 (12.9)	213 (83.6)	2.12 (1.33–3.78)	<0.01	1.66 (0.97–2.84)	0.06
**Age**								
18–24 years	106	6 (5.7)	12 (11.3)	88 (83.0)	1.54 (0.8–2.97)	0.20	NA	NA
25–35 years	204	13 (6.4)	32 (15.7)	159 (77.9)	1.12 (0.66–1.92)	0.68	NA	NA
≥35 years	114	6 (5.3)	22 (19.3)	86 (75.4)	1.00	NA	NA	NA
**Colour/Race**								
White	227	9 (4.0)	30 (13.2)	188 (82.8)	1.00	NA	1.00	NA
Black	54	5 (9.3)	13 (24.1)	36 (66.7)	0.42 (0.22–0.80)	<0.01	0.64 (0.31–1.30)	0.21
Mixed race	138	10 (7.2)	23 (16.7)	105 (76.1)	0.65 (0.39–1.09)	0.10	0.93 (0.53–1.64)	0.81
**Schooling**								
<12 years	104	10 (9.6)	25 (24.0)	69 (66.4)	1.00	NA	1.00	NA
≥12 years	320	15 (4.7)	41 (12.8)	264 (82.5)	2.37 (1.45–3.88)	<0.01	1.90 (1.10–3.29)	0.02
**Gender**								
Male	402	23 (5.7)	62 (15.5)	317 (78.8)	1.00	NA	NA	NA
Trans women	22	2 (8.7)	4 (17.4)	17 (73.9)	0.75 (0.27–1.83)	0.55	NA	NA
**Housing situation**								
Rent or own housing	271	18 (6.6)	43 (15.9)	210 (77.5)	0.77 (0.46–1.27)	0.31	NA	NA
Other (friends/family/public housing)	146	6 (4.1)	21 (14.4)	119 (81.5)	1.00	NA	NA	NA
**Steady partner**								
Yes	239	17 (7.1)	39 (16.3)	183 (76.6)	0.75 (0.47–1.20)	0.23	NA	NA
No	185	8 (4.3)	27 (14.6)	150 (81.1)	1.00	NA	NA	NA
**Sexual behaviour** **in last 3 months**								
**Had sex with client**								
Yes	22	0 (0.0)	6 (27.3)	16 (72.7)	0.80 (0.30–2.12)	0.65	NA	NA
No	402	25 (6.2)	60 (14.9)	317 (78.9)	1.00	NA	NA	NA
**Condomless receptive anal intercourse**								
Yes	191	5 (2.6)	26 (13.6)	160 (83.8)	1.86 (1.14–3.01)	0.01	1.78 (1.08–2.94)	0.02
No	233	20 (8.6)	40 (17.2)	173 (74.2)	1.00	NA	1.00	NA
**Sex with HIV-positive partners**								
Yes	214	13 (6.1)	35 (16.4)	166 (77.6)	0.83 (0.52–1.34)	0.45	NA	NA
No	197	11 (5.6)	27 (13.7)	159 (80.7)	1.00	NA	NA	NA
**Substance use and** **mentalhealth**								
**Binge drinking**								
Yes	249	16 (6.4)	41 (16.5)	192 (77.1)	0.81 (0.50–1.30)	0.39	NA	NA
No	175	9 (5.1)	25 (14.3)	141 (80.6)	1.00	NA	NA	NA
**Any illicit drug use in last 3 months**								
Yes	127	3 (2.4)	20 (15.8)	104 (81.9)	1.38 (0.81–2.34)	0.23	NA	NA
No	296	21 (7.1)	46 (15.5)	229 (77.4)	1.00	NA	NA	NA
**Depression PHQ score**								
PHQ-2 score <3	396	22 (5.5)	60 (15.2)	314 (79.3)	1.00	NA	NA	NA
PHQ-2 score ≥3	27	2 (7.4)	6 (22.2)	19 (70.4)	0.63 (0.27–1.48)	0.29	NA	NA
**STD diagnosis^1^**								
Yes	79	5 (6.3)	14 (17.7)	60 (76.0)	0.83 (0.46–1.47)	0.51	NA	NA
No	339	19 (5.6)	51 (15.0)	269 (79.4)	1.00	NA	NA	NA
**GI symptoms^1^**								
Yes	175	9 (5.1)	28 (16.0)	138 (78.9)	1.02 (0.63–1.63)	0.94	NA	NA
No	245	15 (6.1)	37 (15.1)	193 (78.8)	1 .00	NA	NA	NA

aOR = adjusted odds ratio. ^1^Socio-demogrphics, except “Housing situation”, were assessed at Pre-screening visit. Syphilis was assessed at screening visit and GI symptoms, at week 4. All other variables are from enrolment visit.

**Figure 2.**
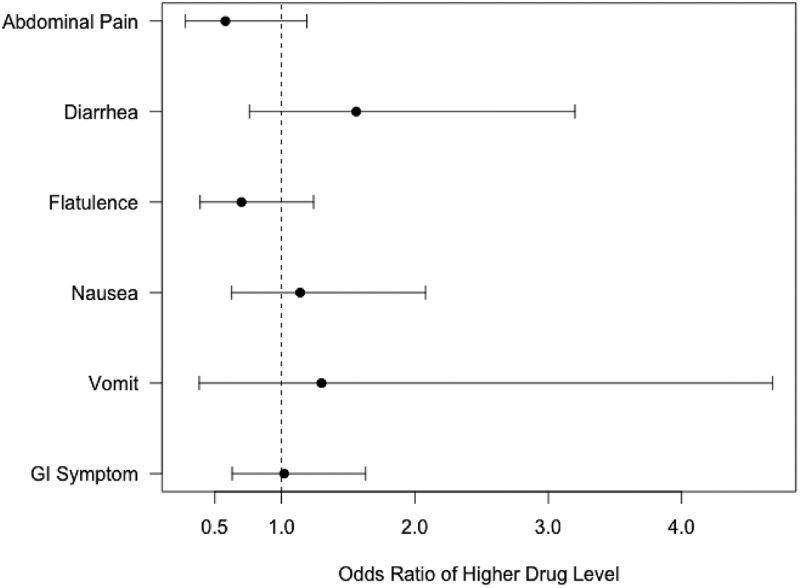
Association of gastrointestinal symptoms and drug level at week-4 after logistic ordinal modelling.

Overall, 42% (178/424) of the participants reported gastrointestinal symptoms at the week 4 visit. None of these symptoms were associated with drug levels (Figure 2).

Of the 25 enrolled TGW, 15 (60.0%) reported current hormone use, mostly oral and/or intramuscular combined contraceptives (11/15, 73.3%). Notably, only 4 of them were using regimens in accordance to TGW specific hormone therapy recommendations [29–31]. Logistic and linear unadjusted regression models showed that hormone use was not associated with higher TFV-DP level or with TFV-DP drug concentration.

## Discussion

PrEP Brasil is the first demonstration project for PrEP naïve MSM and TGW in Latin America and as such, provides a better approximation of what real-world clinical PrEP delivery might look like in Brazil and perhaps other settings in the region. Our results show that interest in PrEP was high, with 60.9% of PrEP uptake. Moreover, even with limited advertising and no outreach, there were a significant number of self-referrals to the study reflecting demand in the community. Eligible but not enrolled individuals were younger, had lower HIV risk perception (also evidenced by their lower rates of previous HIV testing) and had lower PrEP awareness. Efforts to increase HIV risk perception and PrEP awareness are critically needed, especially among younger MSM, as they currently account for nearly 40% of the AIDS cases in the country, with increases of 41.3% (aged 15–19 years) and 25.1% (aged 20–24 years) observed from 2004 to 2015 [17].

Notwithstanding, 25.1% of the enrolled participants were 18–24 years old, which is higher than in the US PrEP demonstration study and in the San Francisco Kaiser PrEP cohort [26,32]. Enrolment of TGW (5.3%), although small, was higher than reported in other studies [26,32]. This is likely related to the concerted and targeted efforts to provide peer education on PrEP to the TGW community in Rio de Janeiro, where the majority of TGW were enrolled. Such activities can play a major role on expanding access to PrEP for TGW. Moreover, in our study, the majority of enrolled participants were self-referred which is higher than in the US Demonstration project (26). This may be accounted by the different regulatory environment, since PrEP was not available in Brazil during the study timeframe.

The high prevalence (0.4%) of HIV acute infection, which is similar but not quite as high as that reported in the iPrEx study [33], suggests that MSM at high risk for HIV acquisition are interested in PrEP. Indeed, PrEP programs stand as a unique opportunity to identify both individuals with undiagnosed acute/early HIV infection who would benefit from immediate antiretroviral therapy (ART) initiation and at risk for HIV acquisition who would benefit from PrEP. PrEP Brasil participants were younger compared to MSM HIV/AIDS cases reported in RJ and SP, suggesting that MSM enrolled in the study were close to the average age of seroconversion. They were also more educated than the Brazilian general population [34] and the MSM population diagnosed with HIV/AIDS [17] from the same cities. Our results show that roughly 30 and 60% of the enrolled participants reported substance use and binge drinking during the prior 3 months, which is also substantially higher than in the Brazilian general population [35,36]. Likely, heavy alcohol and illicit drug use are major drivers of HIV transmission among MSM in our setting as has been shown to be the case elsewhere [37–41]. Furthermore, the high frequencies of condomless receptive anal sex and STDs among the enrolled participants corroborates that those interested in PrEP are at high risk for HIV acquisition.

The present study used intraerythrocytic drug levels measured in DBS to evaluate TDF-based adherence over a 1–2-month horizon [24]. It is an objective biomarker for cumulative adherence, and we were able to perform this assessment for the vast majority of the enrolled cohort (94.2%). Early adherence (at week 4) has been shown to be highly predictive of PrEP persistence [5,11], highlighting the importance of early assessment and adherence support. At week 4, 94.1% (399/424) participants had DBS TFV-DP concentration in the protective range (consistent with ≥2 pills per week), and for 78% participants DBS TFV-DP concentrations were in the highly protective range (≥700 fmol/punch, consistent with ≥4 pills per week), which is estimated to provide of 96–100% protection [2,8]. These drug levels are much higher than those from Brazilian participating sites in iPrEx OLE [11]. Higher levels of adherence in demonstration projects when compared to clinical trials are not uncommon and are partially explained by differing motivations to participate in studies. It may also be related to the growing awareness of PrEP effectiveness in the community [18]. These results are reassuring and provide evidence on the feasibility of PrEP implementation in Brazil.

Moreover, as observed in other clinical trials and demonstration projects, drug levels were higher among those at higher risk of HIV acquisition [1,5,42] reinforcing PrEP’s likely impact and cost-effectiveness. Notably, in our study population, younger age was not associated with lower drug levels, as opposed to iPrEx OLE findings [11]. Despite the high frequency of GI symptoms reported, results showed they have no association with adherence. Also, in agreement with findings from other PrEP demonstration studies neither illicit substance use or binge drinking were associated with TFV-DP levels [2,5] suggesting that these individuals should not be excluded from receiving PrEP due to concerns about non-adherence.

Over 90% of the enrolled TGW showed protective drug concentration levels, with 72.7% achieving highly protective levels. Of note, drug levels between TGW and MSM were not different (*p* = 0.47). Drug levels were not different among those using hormones suggesting that drug interactions may not play a major role, but the small sample of TGW may have limited the analysis’ power to detect an association between use of hormone and drug levels. TGW represent a smaller population than MSM, and have the highest rates of HIV infection worldwide [15] including in Brazil [14,43]. Yet, few TGW have participated in PrEP studies globally [44]. TGW need immediate access to HIV prevention tools, including PrEP, in order to effectively address their devastating HIV burden. Most importantly, PrEP demonstration studies tailored to this population are urgently needed.

The present study has strengths and limitations that should be acknowledged. A major strength was the assessment of drug levels in almost all participants thus providing robust evidence of early adherence. This measure represents cumulative dosing behaviour rather than only recent dosing behaviour, such as that obtained from plasma testing. The week 4 sampling was prior to attainment of steady-state for TFV-DP, so the levels were used to estimate steady state, for interpretation purposes. PrEP persistence [16] was not evaluated in this study and will be reported later upon study completion. Nevertheless, PrEP Brasil participating sites are referral centres highly motivated and well regarded by the community. As such, the high PrEP uptake and/or adherence observed in PrEP Brasil may not be generalizable to other clinical settings. Moreover, our sample was mostly white and highly educated, not reflecting the general population from the cities where the study was conducted; having said that, it is worth noting that the Brazilian HIV/AIDS epidemic remains mostly restricted to large cities, in particular to the two cities where the study was conducted, suggesting appropriateness of the chosen population. Also, the fact that our sample was not probabilistic may hinder statistical generalizability but do provide strong evidence on the reported associations [45]. Although our study enrolled more TGW than most other demonstration projects, they were underrepresented in the sample and mostly enrolled at one of the participating sites. Finally, the fact that our study was implemented at sites that are part of the Brazilian Unified Health System, where the large majority of the Brazilian population will access PrEP when it becomes available in the country, is another noteworthy strength.

## Conclusions

In conclusion, our results show that PrEP for high-risk MSM and TGW can be successfully delivered in the context of the Brazilian Public Health System. The high proportion of participants achieving protective drug levels is encouraging. Moreover, high PrEP early adherence suggests that PrEP use may be an effective strategy to reduce HIV infection among MSM in our setting. Indeed, modelling studies addressing high-risk MSM in high-income settings as well as in Brazil have shown that PrEP is cost-effective in populations at high risk particularly when PrEP efficacy is high [46,47]. Nevertheless, our results suggest that for such benefits to be achieved, strategies to increase risk perception and PrEP awareness among the younger and less educated are needed.
